# MID-LIFE FINANCIAL STRESS AND COGNITIVE AND PHYSICAL PROBLEMS IN OLDER AGE: THE ROLE OF POTENTIALLY MODIFYING FACTORS

**DOI:** 10.1093/geroni/igad104.1251

**Published:** 2023-12-21

**Authors:** Ingemar Kåreholt, Charlotta Nilsen, Deborah Finkel, Shireen Sindi

**Affiliations:** Jönköping University, School of Health and Welfare, Jönköping, Jonkopings Lan, Sweden; Institute of Gerontology, Jönköping, Jonkopings Lan, Sweden; Jönköping University, Jönköping, Jonkopings Lan, Sweden; Karolinska Institutet, Solna, Stockholms Lan, Sweden

## Abstract

Financial stress is an important source of chronic stress and has been associated with cognitive and physical impairments. This study investigates whether midlife financial stress is associated with the combination of cognitive and physical impairment, the role of potentially modifiable factors, and sex differences.

Methods: The Cardiovascular Risk Factors, Aging, and Dementia population-based cohort study from Finland was used (n=1497) (baseline collected 1972-1987, mean age 50 years). There were two late-life re-examinations (mean total follow-up 25 years). Midlife financial stress was measured using two questions on financial situation. Cognitive impairment was based on six cognitive domains. Physical impairment was self-reported. Potential modifying factors investigated were smoking, alcohol, physical activity, cohabiting/not, non-manual work, and sleep disturbances. Sex differences were investigated. We used path analyses with full information maximum likelihood estimation.

Results: Among women and men, midlife financial stress associated with cognitive impairment, physical impairment and their combination. Smoking and sleep disturbances mediated associations between financial stress, physical impairment, and combined impairments. Among men: manual/non-manual work mediated the association to cognitive impairments; cohabitation mediated to cognitive impairment; financial stress was associated with cognitive impairment only among smokers and stress had a stronger association to physical and combined impairments among non-drinkers. Among women, sleep seems to have role in the association between financial stress and cognitive impairment.

Conclusions: Midlife financial stress associates with late-life impairments, and lifestyle/sociodemographic factors may modify these associations. Sex differences were observed. Interventions promoting healthier lifestyle and psychosocial factors may buffer against the deleterious role of financial stress.

